# cUMP elicits interendothelial gap formation during *Pseudomonas aeruginosa* infection

**DOI:** 10.1152/ajplung.00164.2023

**Published:** 2024-07-30

**Authors:** Althea deWeever, Sunita S. Paudel, Chun Zhou, C. Michael Francis, Dhananjay T. Tambe, Dara W. Frank, Ron Balczon, Troy Stevens

**Affiliations:** ^1^Department of Physiology and Cell Biology, University of South Alabama, Mobile, Alabama, United States; ^2^Department of Internal Medicine, University of South Alabama, Mobile, Alabama, United States; ^3^Department of Mechanical, Aerospace and Biomedical Engineering, University of South Alabama, Mobile, Alabama, United States; ^4^Department of Biochemistry and Molecular Biology, University of South Alabama, Mobile, Alabama, United States; ^5^Center for Lung Biology, University of South Alabama, Mobile, Alabama, United States; ^6^Department of Microbiology and Molecular Genetics, Medical College of Wisconsin, Milwaukee, Wisconsin, United States; ^7^Center for Infectious Disease Research, Medical College of Wisconsin, Milwaukee, Wisconsin, United States

**Keywords:** cyclic nucleotide monophosphate, exoenzyme Y, permeability, pneumonia, type 3 secretion system

## Abstract

*Pseudomonas aeruginosa* utilizes a type 3 secretion system to intoxicate host cells with the nucleotidyl cyclase ExoY. After activation by its host cell cofactor, filamentous actin, ExoY produces purine and pyrimidine cyclic nucleotides, including cAMP, cGMP, and cUMP. ExoY-generated cyclic nucleotides promote interendothelial gap formation, impair motility, and arrest cell growth. The disruptive activities of cAMP and cGMP during the *P. aeruginosa* infection are established; however, little is known about the function of cUMP. Here, we tested the hypothesis that cUMP contributes to endothelial cell barrier disruption during *P. aeruginosa* infection. Using a membrane permeable cUMP analog, cUMP-AM, we revealed that during infection with catalytically inactive ExoY, cUMP promotes interendothelial gap formation in cultured pulmonary microvascular endothelial cells (PMVECs) and contributes to increased filtration coefficient in the isolated perfused lung. These findings indicate that cUMP contributes to endothelial permeability during *P. aeruginosa* lung infection.

**NEW & NOTEWORTHY** During pneumonia, bacteria utilize a virulence arsenal to communicate with host cells. The *Pseudomonas aeruginosa* T3SS directly introduces virulence molecules into the host cell cytoplasm. These molecules are enzymes that trigger interkingdom communication. One of the exoenzymes is a nucleotidyl cyclase that produces noncanonical cyclic nucleotides like cUMP. Little is known about how cUMP acts in the cell. Here we found that cUMP instigates pulmonary edema during *Pseudomonas aeruginosa* infection of the lung.

## Graphical abstract

**Figure:**
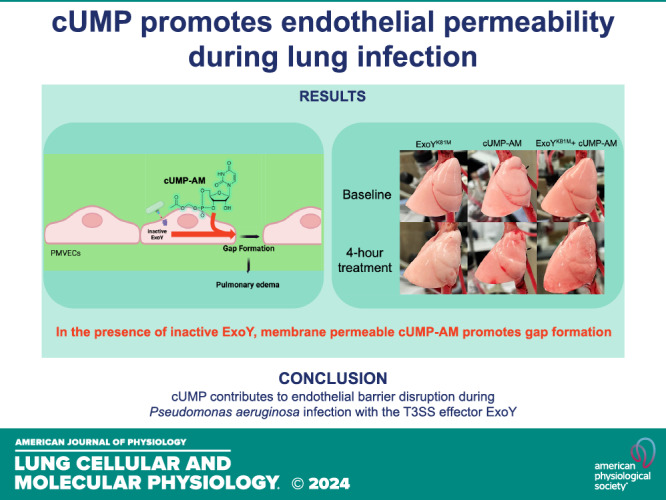

## INTRODUCTION

*Pseudomonas aeruginosa* is an opportunistic pathogen that is a common cause of nosocomial infections, and one that is particularly problematic in immunocompromised patients in the intensive care unit (ICU) (1, 2). Although *P. aeruginosa* is a cause of pneumonia, it is also a direct cause of acute respiratory distress syndrome (ARDS), and it can evolve into sepsis (3, 4). Mortality rates in patients with ARDS remain unacceptably high, that is, ∼30–40% (5). In addition, survivors of this critical illness experience high healthcare costs with the emergence of life-altering complications post-discharge (3, 6–8). Indeed, the impact of critical illness extends beyond the hospital stay. Emerging evidence indicates that survivors exhibit higher morbidity and mortality rates in the aftermath of their critical illness (9–12). For example, some studies report mortality rates as high as 50% after discharge from the ICU (13, 14). Chronic end-organ dysfunction is common among survivors of the ICU, including high rates of stroke, myocardial infarction, neuromuscular weakness, and neurocognitive dysfunction, among others (15–19).

The mechanisms of protracted end-organ dysfunction in the aftermath of critical illness are poorly understood. However, preclinical studies indicate that the bacterial virulence arsenal plays a key role in determining the host response to infection, and consequently the severity of end-organ dysfunction (20). For example, the *P. aeruginosa* type three secretion system (T3SS) and its effectors represent virulence determinants that negatively impact host outcomes (21). Four exoenzymes are introduced through the T3SS needle directly into the host cell cytoplasm, evading immune detection by the innate immune system. Once inside cells, the exoenzymes acquire a tertiary conformation and bind to the mammalian cofactor necessary for enzymatic activity. Exoenzymes S and T utilize 14-3-3 proteins as cofactors and on activation acquire ADP-ribosyltransferase activity, especially while remodeling the actin cytoskeleton (22). By contrast, exoenzyme U utilizes ubiquitinated proteins as a cofactor and on activation is a phospholipase that degrades cellular membranes (23). Exoenzyme Y, the most recently identified T3SS effector, utilizes filamentous actin as a cofactor and upon activation acquires promiscuous nucleotidyl cyclase activity, producing cGMP, cAMP, cCMP, and cUMP (24–26). Together, the intracellular action of these enzymes elicits a host cell response that contributes to end-organ dysfunction.

The *P. aeruginosa* exoenzyme Y (ExoY) has been linked to end-organ dysfunction and poor patient outcomes (20). It is found in ∼ 90% of clinical isolates from patients with nosocomial pneumonia, illustrating its high prevalence and highlighting the importance of better resolving its contribution to end-organ dysfunction (20). In lung endothelium, the ExoY-induced cyclic nucleotides activate protein kinase A, which phosphorylates microtubule-associated protein tau leading to its dissociation from microtubules. Dissociation of tau from microtubules results in their reorganization and breakdown, which causes interendothelial gap formation, disrupts the alveolar-capillary barrier, and promotes pulmonary edema (27–31). Hyperphosphorylated tau assembles into oligomers and/or cytotoxic variants that are found outside the cell in in vitro experiments and the bronchoalveolar lavage fluid, blood, heart, cerebrospinal fluid, and brain parenchyma, including the hippocampus, of infected animals and human subjects (32, 33). The infection-elicited tau variants are cytotoxic and contribute to end-organ dysfunction (30, 34–38). ExoY therefore promotes the generation of cytotoxic tau variants that contribute to acute and chronic outcomes following infection.

ExoY generates both purine (i.e., canonical) and pyrimidine (i.e., noncanonical) cyclic nucleotides, which can activate PKA (28). However, the role(s) of noncanonical cyclic nucleotides, like cUMP, are poorly understood. Here, we examine whether cUMP contributes to the virulence induced by ExoY, in the absence of other canonical (i.e., cAMP and cGMP) and noncanonical (i.e., cCMP) cyclic nucleotides. Our results reveal an important intracellular role for cUMP in promoting endothelial cell barrier disruption.

## MATERIALS AND METHODS

### Pulmonary Microvascular Endothelial Cell Culture

Rat pulmonary microvascular endothelial cells (PMVECs) were sustained in culture, as described elsewhere. PMVECs were propagated in Dulbecco’s modified Eagle’s medium (DMEM) with 10% heat-inactivated fetal bovine serum (Atlanta Biologicals #S11550H) and 1% penicillin-streptomycin (Invitrogen #15140-122) and incubated at 37°C in 21% O_2_ and 5% CO_2_. Cells were routinely seeded into six-well plates at 1.5 × 10^5^ cells per well and grown to confluence over ∼3 days. Once confluence was established, cells were infected with bacteria with or without cUMP-AM for the indicated periods of time (see *Endothelial Infection*).

### Bacterial Strains and Growth Conditions

PMVECs were infected with mutant *P. aeruginosa* strains that were derived from PA103. The ExoY^+^ mutant possesses a functional type three secretion system (T3SS). In this mutant, exoenzymes T and U have been deleted and exoenzyme Y has been introduced in a plasmid (PA103Δ*exoUexoT*::Tc pUCP*ExoY+*; ExoY^+^). This strain was described previously (26). In addition, ExoY^K81M^ (PA103Δ*exoUexoT*::Tc pUCP*ExoY*^K81M^;ExoY^K81M^) is a mutant of ExoY that is enzymatically inactive due to a point mutation at residue 81, where lysine (K) is converted to methionine (M) (26).

Using aseptic conditions, bacteria were struck from frozen stocks and grown overnight on Vogel-Bonner minimal salt media with 400 μg/mL of carbenicillin (Sigma #C1389). Bacteria were scraped from the agar with an inoculation loop and resuspended in phosphate buffered solution (PBS, Invitrogen #10010-023) to an optical density of 0.25 at 540 nm, which equals 2 × 10^8^ colony forming units/mL. Hanks’ balanced salt solution (HBSS) with Ca^2+^ and Mg^2+^ (Invitrogen #14025-134) was used to dilute the bacterial suspensions to attain a multiplicity of infection (MOI) of 20:1.

### Endothelial Infection

PMVECs were seeded in six-well cell culture dishes as described in *Pulmonary Microvascular Endothelial Cell Culture* and grown to 100% confluence. To obtain cell counts, cells were washed with PBS, trypsinized into single-cell suspensions, media was added to deactivate trypsin, and cell counts were obtained using a Countess Cell Counter II (Invitrogen). HBSS was placed on cells for 15 min and then washed. The cells were infected with ExoY^+^ (PA103Δ*exoUexoT*::Tc pUCP*exoY*) or ExoY^K81M^ (PA103Δ*exoUexoT*::Tc pUCP*exoY^K81M^*), with or without uridine-3′,5′-cyclic monophosphate, acetoxymethyl ester (cUMP-AM at 1, 10, or 100 µM; Biolog #U 012), at a MOI of 20:1. For control experiments, PMVECs were treated likewise in the presence or absence of ExoY^K81M^ with either 100 µM uridine- 3′,5′- cyclic monophosphate sodium salt (cUMP, no-AM group; Biolog #U 001), 33 µM phosphate tris(acetoxymethyl)ester (PO_4_-AM_3_, control for -AM group; Biolog #P 030), or 100 µM guanosine-3′,5′-cyclic monophosphate, acetoxymethyl ester (cGMP-AM; Biolog #G 002). The cells were infected for 6 h. For vehicle controls, HBSS with 0.1% DMSO was placed on cells for 6 h. For growth curves and time-lapse experiments, cells were infected for 4 h and cells were treated with one concentration of cUMP-AM, 25 µM.

### Gap Analyses

A gap analysis was performed using a custom Image J macro as published previously (39). First, the image contrast was adjusted to 15% saturated pixels. The “subtract background” command was used on the contrast-adjusted images to obtain a high-contrast image of both the cell and gap area within the image. This image was then subtracted from the original image using the image calculator “AND” function. The threshold function was then used to convert the resulting image to black (gaps) or white (cells) mask. The “binary erode” function was used to remove noise from the image. “Area fraction” measurement was used to measure the ratio of black to white pixels in the black and white image. Fractional areas from each treatment group were plotted as a percent of the maximal gap area.

### Growth Curves

After 4-h infection, PMVECs were extensively washed with PBS and grown in DMEM media with 10% serum in the presence of penicillin and streptomycin, along with 5 µg/mL gentamicin and 5 µg/mL chloramphenicol to remove bacteria. In total, 24 h later, cells were seeded at a density of 1 × 10^5^ and allowed to grow to confluency over 4 days. Every 24 h, images were taken then cells were trypsinized and counted with a Countess Cell Counter (Thermo Fisher).

### Time-Lapse Microscopy for Cellular Morphology and Movement Analysis

Cellular morphology (size and circularity) and speed were evaluated postinfection. As mentioned in the *Endothelial Infection* section, cells were extensively washed with PBS and then a cell culture medium with 10% serum and antibiotics was placed on the cells to kill and inhibit bacterial growth during microscopy. Image acquisition was performed at 20× with a Nikon T2-Eclipse microscope equipped with a stage-top environmental control chamber to provide a physiological setting (37°C, 21% O_2_, 5% CO_2_, and 74% N_2_) for the cells as previously published (40). Images were acquired at 5-min intervals for the time of the infection and 18–20 h afterward. Cellular morphology was calculated using ImageJ-based custom software: Integrative Toolkit to Analyze Cellular Signals (iTACS) as described here (41, 42). In short, the software performed cellular segmentation of each individual cell to allow the morphological characteristics of the cells to be determined. Shape and size were given by area (*A*) and circularity [*C* = 4π(*A*/*P*^2^)] where *P* is the perimeter. A value of 1 signifies a perfectly circular cell, whereas lower values indicate the elongated cells or other noncircular forms. Also, cell segmentation allowed cellular movement and speed analysis via the particle image velocimetry (PIV) plugin of ImageJ. Each individual cell was tracked per image to provide information regarding cellular movement.

### Isolated Lung Experiments

Experimentation with animals was approved by the Institutional Animal Care and Use Committee of the University of South Alabama, and conducted according to the “Guide to the Care and Use of Laboratory Animals.” Rats were anesthetized using Nembutal (65 mg/kg body wt). Once a surgical plane was achieved, as defined by the absence of a withdrawal reflex following toe and tail pinch, rats were intubated and ventilated, a sternotomy was performed, and pulmonary artery and left ventricle/atrium catheters were placed. The heart and lungs of adult male and female Sprague–Dawley rats, weighing ∼250 g, were removed en bloc and suspended in a humidified chamber where mechanical ventilation and flow were established. Rat lungs were perfused with buffer (in mmol/L: 119.0 NaCl, 4.7 KCl, 1.17 MgSO_4_, 1.18 KH_2_PO_4_, 23 NaHCO_3_, and 5.5 glucose) containing 4% bovine serum albumin and physiological (2.2 mmol/L) CaCl_2_, plus 6% autologous whole blood. After an initial isogravimetric period a baseline filtration coefficient (*K*_f_) was measured. The filtration coefficient was calculated as the rate of weight gain obtained 13–15 min following a 10 cm H_2_O increase in pulmonary venous pressure, normalized per 100 g of the predicted wet lung weight. Pulmonary artery and venous pressures and lung weight were measured continuously, and double occlusion pressure was measured under stop-flow conditions. Following the baseline *K*_f_, bacteria were instilled into the trachea at a concentration of 75 × 10^6^ CFU. At 1 h after the instillation, 25 µM cUMP-AM was injected into the pulmonary circulation through the pulmonary artery catheter. *K*_f_ was measured again at 2 and 4 h after bacterial infection to assess alveolar-capillary permeability.

### Statistical Analysis

GraphPad Prism 9.0 software package was used for statistical analyses. Kruskal–Wallis with Dunn’s or Benjamini–Hochberg post hoc test, Mixed-effects analysis with Tukey’s post hoc, or two-way ANOVA with Tukey’s post hoc were used to test for statistical significance as applicable. Significance is denoted as any *P* value ≤ 0.05.

## RESULTS

### Membrane Permeable Cyclic Uridine Monophosphate Promotes Interendothelial Gap Formation during Infection with ExoY^K81M^

**Figure 1.**
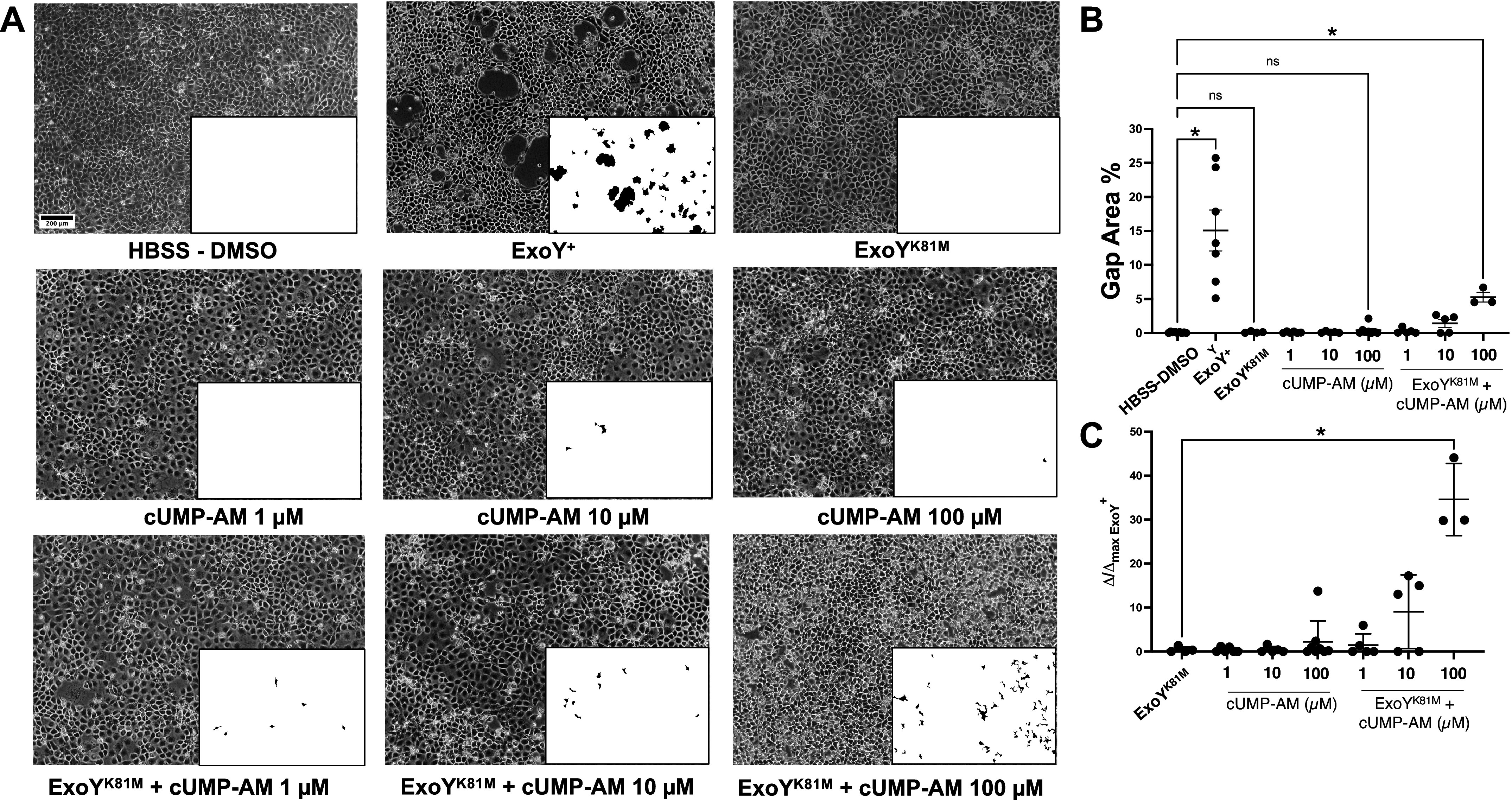
cUMP-AM stimulates interendothelial gap formation during ExoY^K81M^ infection. *A*: pulmonary microvascular endothelial cells were treated with HBSS with 0.1% DMSO as a control, cUMP-AM alone (1, 10, or 100 µM), or infected for 6 h at a 20:1 multiplicity of infection (MOI) with ExoY^+^ or ExoY^K81M^ with or without cUMP-AM (1, 10, or 100 µM). *B*: gap analyses of images taken after infection were analyzed by a custom macro in ImageJ. Kruskal-Wallis with an uncorrected Dunn’s post hoc test, means ± SD; *P ≤ 0.05; ns, not significant. *C*: gap analyses are represented with each data point as a percentage of the average ExoY^+^ gap formation. Kruskal–Wallis with Benjamini–Hochberg post hoc test, means ± SD, *P ≤ 0.05. Images are representative of 3–7 experiments, taken with ×10 objective, scale bar: 200 μm, inset: black-white gap analysis map where black regions indicate gapping.

ExoY^+^ produces canonical (e.g., cGMP and cAMP) and noncanonical (e.g., cUMP and cCMP) cyclic nucleotides during infection, which cause interendothelial cell gap formation (26, 31, 43–48). The cytosolic cAMP, and to a lesser extent cGMP produced by ExoY^+^ contribute to gap formation during infection (28). Here, we sought to clarify whether cUMP also contributes to interendothelial gap formation elicited by ExoY^+^. PMVECs were infected with *P. aeruginosa* possessing catalytically active ExoY^+^ and the catalytically inactive mutant ExoY^K81M^. Consistent with prior studies (30, 49), ExoY^+^ elicited a time-dependent endothelial retraction that was uniformly distributed across the monolayer (Fig. 1). By stark contrast, ExoY^K81M^ did not elicit endothelial retraction or gap formation. Thus, the enzymatic activity of ExoY^+^ is necessary for these *P. aeruginosa* mutants to disrupt endothelial cell barrier integrity, over the time course of our experiment.

To address the role of cUMP in interendothelial cell gap formation, we treated PMVECs with various concentrations (1, 10, or 100 µM) of membrane permeable cUMP-AM for 6 h and assessed barrier integrity. None of the cUMP-AM concentrations tested promoted significant gap formation. (Fig. 1, *A* and *B*). Therefore, cUMP-AM does not promote interendothelial gap formation in the absence of infection.

To evaluate whether cUMP contributes to gap formation during a *P. aeruginosa* infection, we treated PMVECs with ExoY^K81M^ in combination with ascending concentrations of cUMP-AM (Fig. 1). In the presence of ascending concentrations of cUMP-AM, ExoY^K81M^ promoted interendothelial gap formation. ExoY^K81M^ accompanied by 100 µM cUMP-AM recapitulated ∼35% of the gap area that was generated by active ExoY^+^ (Fig. 1*C*). In control studies, we interrogated the effects of cUMP without the AM group and the AM group alone, with or without ExoY^K81M^. Neither cUMP nor the free-AM group potentiated the ExoY^K81M^-induced permeability (Supplemental Fig. S1). We also examined the effect of cGMP-AM and found that it did not potentiate the ExoY^K81M^-induced permeability response like cUMP-AM. (Supplemental Fig. S1). Therefore, this evidence supports the idea that cUMP can contribute to interendothelial gap formation during ExoY^+^ infection.

**Figure 2.**
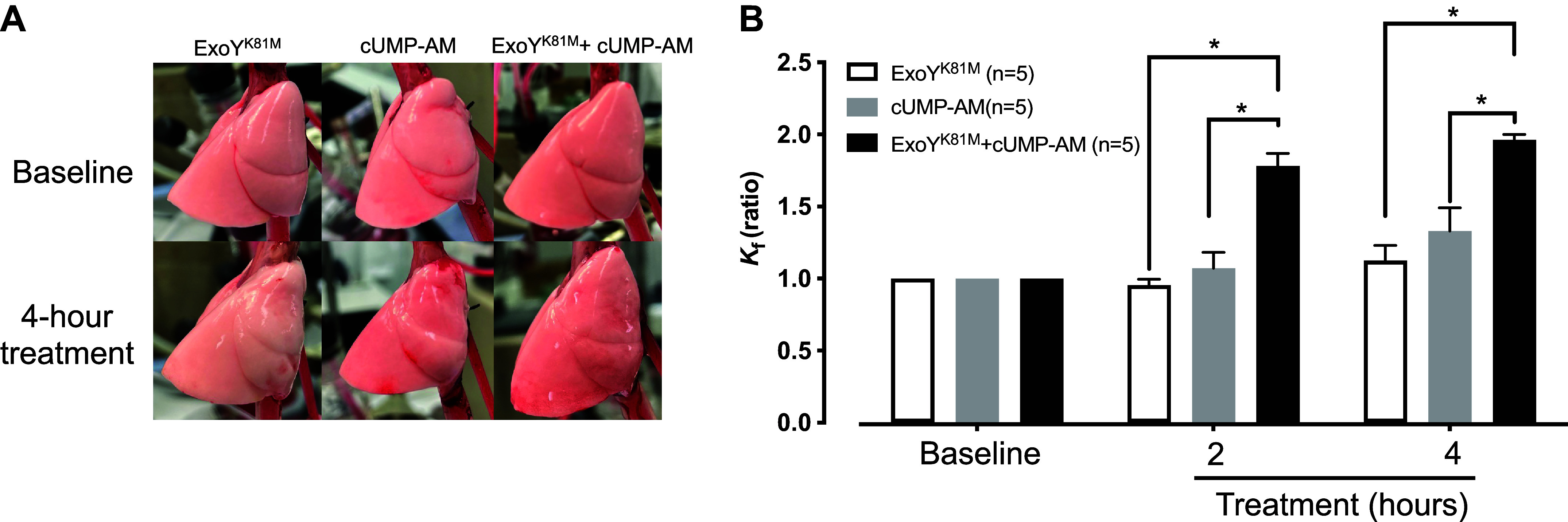
Cyclic UMP promotes endothelial permeability during ExoY^K81M^ infection. *A*: gross inspection of the isolated perfused lung infected with ExoY^K81M^ in the presence and absence of cUMP-AM (25 µM), before and after infection. Note edematous patches in the presence of cUMP-AM. Images are representative of five separate experiments. *B*: comparison of filtration coefficient (*K*_f_) values as an indicator of permeability, taken at 0, 2, and 4 h. Two-way ANOVA with Tukey post hoc test, means ± SE, *P ≤ 0.05.

We next examined whether cUMP-AM promotes pulmonary edema in an isolated perfused lung preparation. Following heart and lung isolation *en bloc*, a 15-min isogravimetric period was established and then a baseline filtration coefficient measurement was performed. ExoY^K81M^ was delivered through the trachea, and the filtration coefficient was measured 2 and 4 h later. The introduction of ExoY^K81M^ into the airways did not increase the filtration coefficient over the 4-h time course (Fig. 2). We then introduced ExoY^K81M^ into the airways and 1 h later delivered cUMP-AM into the circulation. Cyclic UMP-AM in combination with ExoY^K81M^ increased the filtration coefficient approximately twofold (Fig. 2). By gross inspection, lungs treated with the ExoY^K81M^ and cUMP-AM combination displayed widespread edematous patches visible on the lung’s surface. By contrast, permeability was not increased in the lungs that were infected with ExoY^K81M^ in the absence of cUMP-AM. Cyclic UMP-AM treatment alone did not increase permeability. Hence, at the concentration tested, cUMP-AM was not sufficient to increase permeability, yet in combination with infection with ExoY^K81M^, cUMP-AM led to endothelial barrier disruption and pulmonary edema.

### Cyclic UMP-AM Does Not Impair Endothelial Cell Recovery following Infection

**Figure 3.**
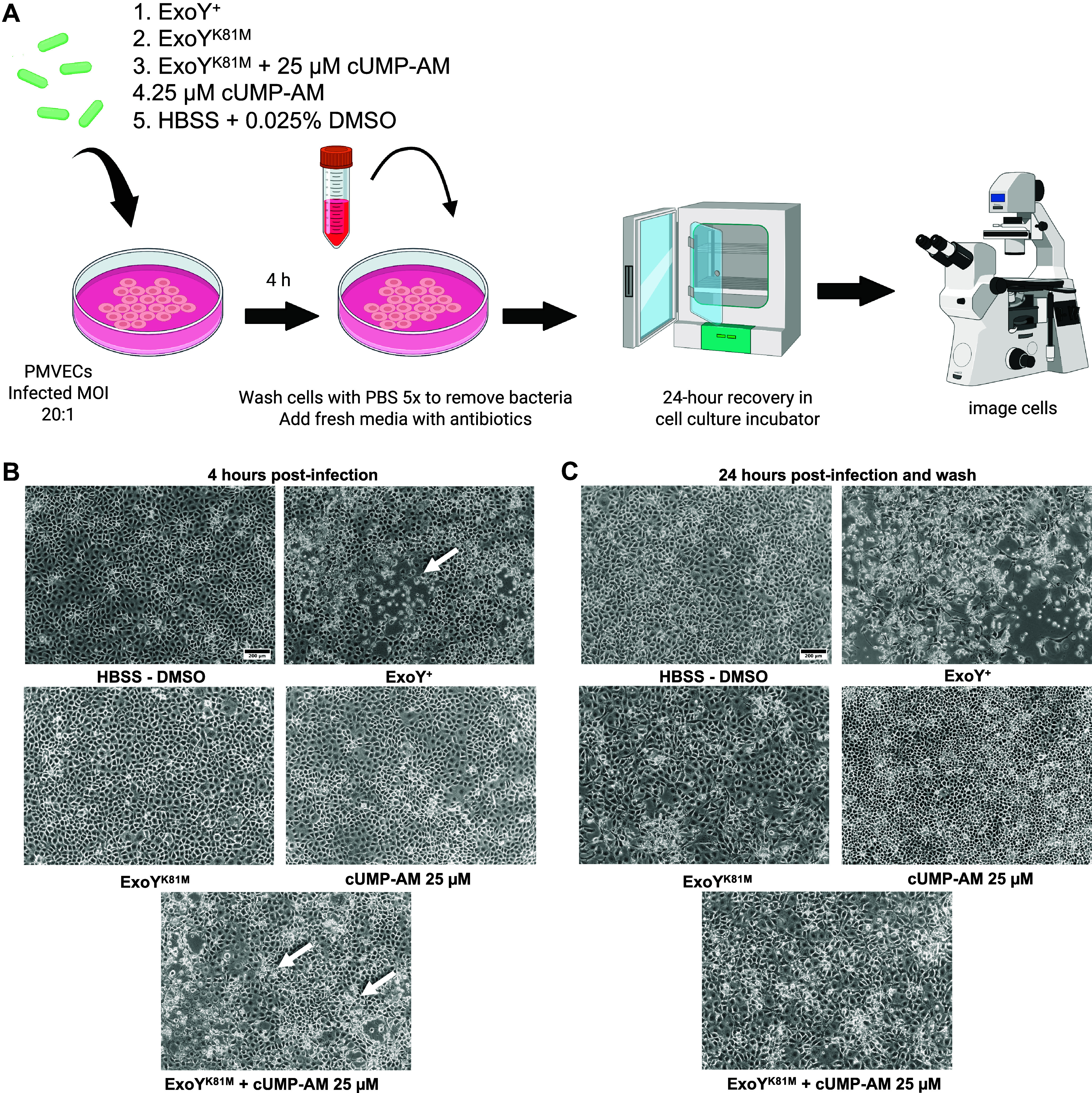
ExoY^+^-infected cells do not reseal infection-induced gaps 24 h postinfection. *A*: pulmonary microvascular endothelial cells (PMVECs) were infected with either ExoY^+^, ExoY^K81M^, ExoY^K81M^ with 25 µM cUMP-AM, all at a multiplicity of infection (MOI) of 20:1, or alternatively treated with 0.025% DMSO or 25 µM cUMP-AM without bacteria. Treatments were for 4 h. Following the treatment, cells were washed and a fresh medium with additional antibiotics was added. Cells were allowed to recover for 24 h and then imaged to assess their recovery. *B*: in total, 4-h infection is sufficient to promote interendothelial cell gap formation following ExoY^+^ and ExoY^K81M^ + 25 µM cUMP-AM infection. *C*: ExoY^+^-infected cells did not recover postinfection, whereas the ExoY^K81M^ + 25 µM cUMP-AM-treated cells did reseal. Images are representative of 5–10 experiments of each experimental group. Images were taken using a ×10 objective. The scale bar represents 200 µm. [Figure created with Biorender.com.]

Previous studies revealed that 6-h infection of PMVECs with ExoY^+^, but not with ExoY^K81M^, generates cell gaps in confluent PMVEC monolayers and compromises repair days after the infection (50). Here, we tested whether 4-h infection similarly impairs the resealing of the interendothelial cell barrier (Fig. 3*A*). Over this shortened time course, gaps formed in monolayers infected with ExoY^+^, but not with ExoY^K81M^ (Fig. 3*B*). Bacteria were then removed by vigorous washing, and fresh medium with 10% serum and antibiotics were added. In total, 24-h postinfection the cells were imaged to assess whether the PMVEC monolayers resealed (Fig. 3*C*). After ExoY^+^ infection, cells failed to reseal, and instead, they became elongated. A population of the cells appeared to die 24 h postinfection; cell counting confirmed that the number of live cells greatly diminished 24 h after ExoY^+^ infection (data not shown). In total, 24 h following ExoY^K81M^ infection some gaps appeared in the monolayer; however, the cells still appeared healthy. Nonetheless, cell counts following the ExoY^K81M^ infection were decreased (data not shown). These findings support the idea that ExoY^+^, but not ExoY^K81M^, most prominently reduces endothelial cell repair following infection.

We next sought to determine whether cUMP can impede endothelial repair after 4 h of ExoY^+^ infection. To test this idea, confluent monolayers of PMVECs were treated with 25 µM cUMP-AM in the presence or absence of ExoY^K81M^ (Fig. 3, *B* and *C*). After 4 h of treatment, cUMP-AM did not cause endothelial cell gap formation, but in combination with ExoY^K81M^, gaps formed, and the cells rounded. Cells were allowed to recover for 24 h postinfection. With cUMP-AM treatment, the cells appeared healthy and cell numbers did not decrease. Unlike the ExoY^+^-treated cells, cells treated with the ExoY^K81M^ and cUMP-AM combination were able to reseal (Supplemental Video S1). These cells appeared larger than those treated with cUMP-AM alone, and there was a reduction in the total number of cells recovered 24 h postinfection (data not shown; see also Fig. 5*C*). Therefore, although cUMP-AM promotes gap formation in ExoY^K81M^-infected cells, it does not prevent the monolayer from repairing in the aftermath of the infection.

### Membrane Permeable cUMP-AM Partially Rescues Impaired Proliferation following ExoY^K81M^ Infection

**Figure 4.**
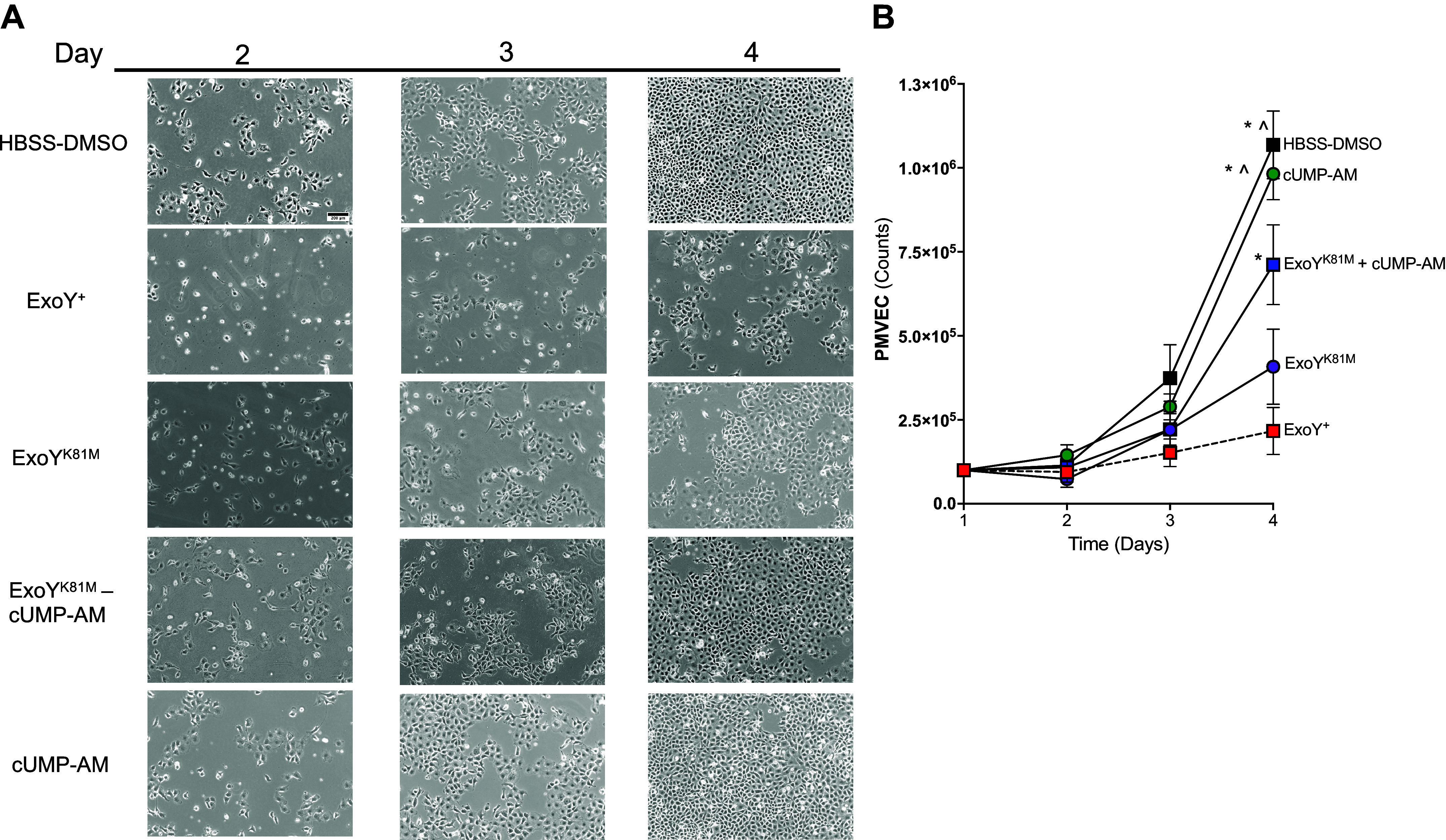
cUMP-AM does not recapitulate the stunted proliferation that is induced by ExoY^+^ infection. Pulmonary microvascular endothelial cells (PMVECs) were infected with ExoY^+^, ExoY^K81M^, ExoY^K81M^ + 25 µM cUMP-AM, 25 µM cUMP-AM, or treated 0.025% DMSO in HBSS, washed extensively with PBS, then treated with medium in the presence of 10% serum and antibiotics to remove bacteria. After 24 h PMVECs were seeded at 1 × 10^5^ cells and allowed to grow to confluence. *A*: images of cells were taken every 24 h until control HBSS-DMSO-treated PMVECs were confluent as seen on *day 4*. All treatments reached near confluency by *day 4* except for ExoY^+^. Cyclic UMP-AM treatment did not stunt growth but surprisingly rescued proliferation post-ExoY^K81M^ infection. *B*: cells were counted every day after 24 h of growth, Two-way ANOVA with Tukey’s post hoc test, means ± SE, *P ≤ 0.05 vs. ExoY^+^-infected cells. Mixed effects analysis with Tukey’s post hoc test, ^*P* ≤ 0.05 vs. *day 1* of growth curve. Images are representative of five separate experiments, taken with ×10 objective, scale bar: 200 μm.

ExoY^+^ reduces cell proliferation in the postinfection period (50). To assess whether cUMP contributes to this phenomenon, PMVECs were infected with either ExoY^+^ or ExoY^K81M^ for 4 h, cells were reseeded, and a growth curve was performed (Fig. 4*A*). As previously reported (49), ExoY^+^-infected cells failed to proliferate and did not reach confluency by *day 4* (doubling time = 4 days), in stark contrast to the control cells (doubling time = 0.5 days) (Fig. 4*B*). Cells infected with ExoY^K81M^ proliferated, but they also failed to achieve confluency within 4 days (doubling time = 0.8 days). Therefore, ExoY intoxication injures endothelial cells, preventing cellular recovery and proliferation postinfection.

To assess whether cUMP contributes to the ExoY^+^-dependent impairment in endothelial proliferation, we tested proliferation after cUMP-AM treatment with and without ExoY^K81M^ infection. Cells treated with cUMP-AM alone grew rapidly and became confluent by *day 4* (doubling time = 0.6 days); cell counts were not different between control and cUMP-AM-treated cells. Cells treated with cUMP-AM during the ExoY^K81M^ infection grew rapidly, and they were nearly confluent by *day 4* (doubling time = 0.7 days) (Fig. 4, *A* and *B*). These results indicate that although cUMP-AM promotes gap formation and permeability during infection, it does not lead to a protracted impairment of proliferation.

### Cyclic UMP-AM Has Little to No Effect on Cell Speed and Spread Area with or without ExoY^K81M^

**Figure 5.**
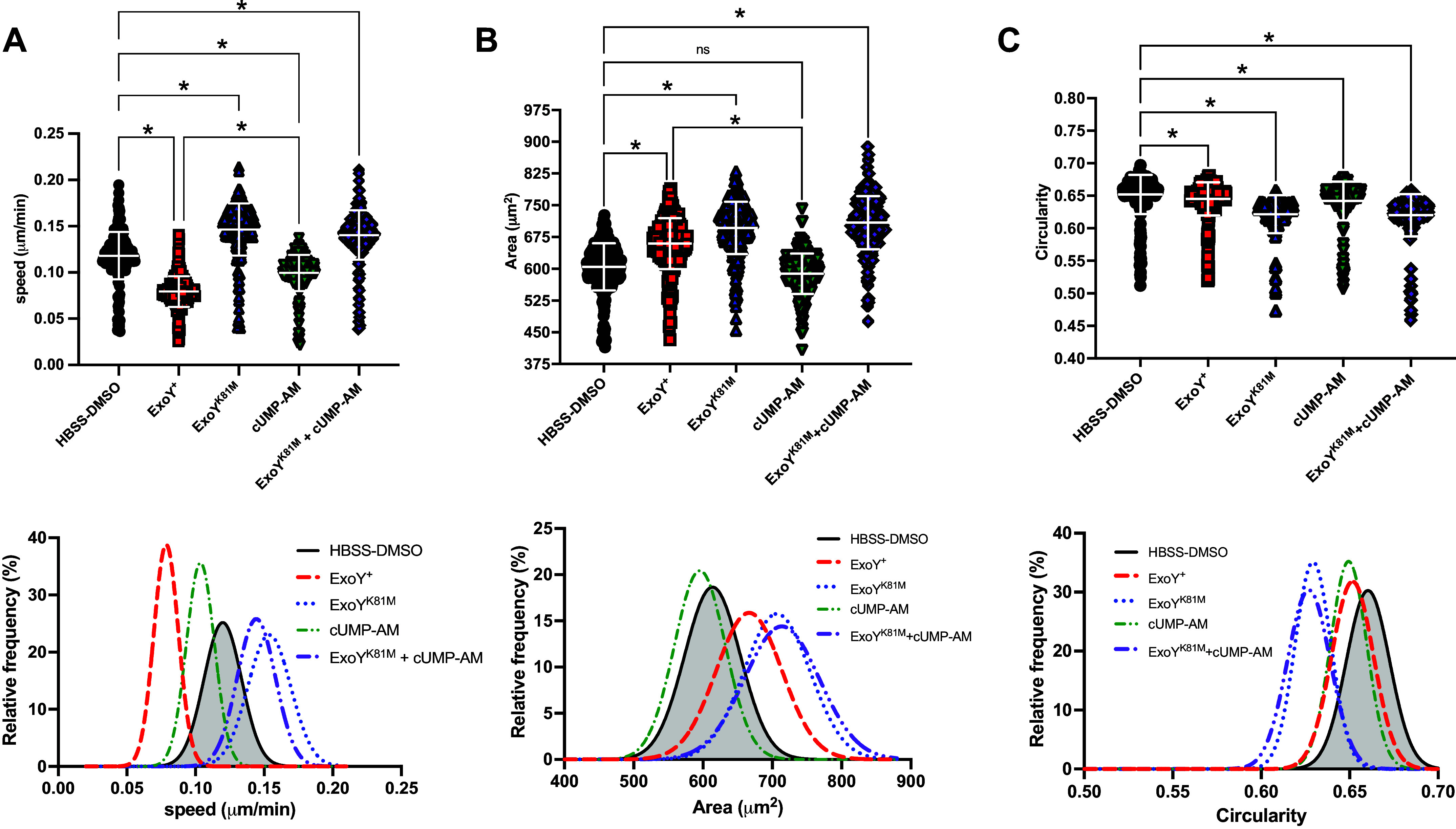
Cell motility is reduced following ExoY^+^ infection and cUMP-AM treatment. Time-lapse microscopy was performed using a Nikon T2-eclipse microscope, where images were captured every 5 min for 12 h while cells were maintained at 37°C, 21% O_2_, 5% CO_2_, and 74% N_2_ using an environmental chamber. Images taken from the first and last 2 h of the 12-h video were analyzed; 225 cells from each experiment were compared for analysis. *A*: ExoY^+^-treated cells exhibited decreased, whereas ExoY^K81M^-treated cells exhibited increased, cellular speed. Kruskal–Wallis test with Dunn’s post hoc, means ± SD, *P ≤ 0.05. Frequency histograms fitted with Gaussian curve, goodness of fit, *r*^2^ = 0.93–0.97 *B*: area of individual cells postinfection. Spread area was greatest in ExoY^K81M^-treated cells, followed by ExoY^+^-treated cells. cUMP-AM treated cells exhibited the smallest spread area. Kruskal–Wallis test with Dunn’s post hoc, means ± SD, *P ≤ 0.05. Frequency histogram fitted with Gaussian curve, goodness of fit *r*^2^ = 0.98–0.99. Five videos of each treatment were analyzed and compared. *C*: circularity of cells postinfection, where 1 represents a perfect circle and lower values indicate elongation. ExoY^+^-treated cells were more circular than ExoY^K81M^-treated cells. Kruskal–Wallis test with Dunn’s post hoc, means ± SD, *P ≤ 0.05. Frequency histogram fitted with Gaussian curve, goodness of fit *r*^2^ = 0.94–0.98.

ExoY^+^ infection leads to diminished endothelial cell motility (50). Here, we sought to quantify cell speed postinfection, and further, to characterize morphological changes cells exhibit during the recovery period. To do this, we used time-lapse microscopy to image the cells at 5-min intervals over 12 h and analyzed cell movements from the first and last 2 h of the experiment (Fig. 5). In controlled conditions, PMVECs move at a speed of ∼0.12 μm/min, have an approximate 600 μm^2^ spread area, and have a circularity of ∼0.65 (Fig. 5; Supplemental Video S2). Compared with this, the ExoY^+^ treatment reduced endothelial speed by 40%, increased cellular spread area by more than 10%, and slightly reduced circularity (Fig. 5; Supplemental Video S3). By contrast, ExoY^K81M^ treatment increased endothelial speed by 20%, increased cellular spread area by 16%, and reduced circularity by 5% (Fig. 5; Supplemental Video S4). Thus, the enzymatic activity of ExoY is essential for the reduction in cell speed exhibited after infection.

Compared with the control condition, cUMP-AM treatment reduced cellular speed by 20% and reduced cellular spread area and circularity slightly (Fig. 5; Supplemental Video S5). The outcomes of a combined cUMP-AM and ExoY^K81M^ treatment were like those produced by ExoY^K81M^ treatment (Fig. 4; Supplemental Video S6). Thus, cUMP does not likely account for the reduction of cell motility post ExoY^+^ infection.

## DISCUSSION

Exoenzyme Y is a T3SS effector of *P. aeruginosa*. It is a promiscuous nucleotidyl cyclase that enzymatically produces both purine and pyrimidine cyclic nucleotides. Cyclic AMP, cGMP, and cUMP initiate signaling pathways that instigate interendothelial barrier disruption, pulmonary edema, and vascular dysfunction, as evidenced by compromised proliferation and vascular repair following infection (50). Prior studies revealed that the purine cyclic nucleotides cAMP, and to a lesser extent cGMP, promote endothelial barrier disruption and pulmonary edema. However, whether cUMP contributes to endothelial cell barrier disruption and/or disrupts processes leading to vascular repair in the wake of infection is unknown. Our studies demonstrate that cUMP-AM in the presence of ExoY^K81M^ promotes interendothelial gap formation and induces pulmonary edema yet does not impair cell motility and endothelial proliferation. These data support the idea that cUMP contributes to the acute processes that disrupt the alveolar-capillary membrane during infection.

We revealed that membrane-permeable cUMP-AM stimulates interendothelial gap formation and pulmonary edema during infection when inactive ExoY^K81M^ is present. These studies highlight the injurious role of cUMP in a clinically relevant pathology (47, 51). This effect of cUMP required the co-presence of ExoY^K81M^; cUMP-AM alone did little to intensify permeability. Our present studies were not designed to determine mechanisms of synergy between cUMP and ExoY^K81M^. However, cUMP activates a PKA signal that may be coupled to microtubule reorganization, whereas ExoY^K81M^ binds f-actin and competes with Arp2/3 binding to inhibit branching. Future studies will examine whether this combination of cytoskeletal targets is sufficient to disrupt endothelial barrier integrity.

During infection with ExoY-competent *P. aeruginosa* mutants, cUMP begins to accumulate in the cell within 2–3 h, and it achieves high and sustained concentrations for at least 6 h (31). How cUMP is compartmentalized within the cell during infection is poorly understood. cUMP may be degraded by phosphodiesterases or extruded by multidrug-resistant proteins (MRPs) (52). Phosphodiesterases 3A, 3B, and 9 have been reported to hydrolyze cUMP in vitro; however, the phosphodiesterase responsible for cUMP degradation in PMVECs is unknown (53–55). MRP4 and 5 are expressed in PMVECs (data not shown), and these proteins transport cUMP from cells (56). Extrusion from the cell may be a major elimination mode of cUMP, as high cUMP concentrations have been resolved in urine and feces after ExoY^+^ infection (57).

We sought to determine whether cUMP contributes to the decrease in vascular repair following ExoY intoxication. Our studies found no prolonged, negative impact of the elevation of cUMP using the AM form to deliver cUMP. Actin remodeling plays a key role in cell migration. ExoY competes with Arp2/3 binding to filamentous actin, which is necessary for its branching (24). Both ExoY and ExoY^K81M^ bind to filamentous actin, yet the K81M mutation renders the enzyme inactive (26, 58). As ExoY^+^ but not ExoY^K81M^ disrupted endothelial cell migration, it is unlikely that direct binding to filamentous actin is the cause of impaired endothelial migration. Future studies will be required to assess other cAMP- and/or cGMP-driven events that underlie the decrease in endothelial migration following infection.

In in vitro wound healing assays, cells fill the available open spaces by spreading, migrating, and proliferating. In these endothelial assays, 75% of the wound closure is attributed to cellular spreading and migration, and 25% to cellular proliferation (59). As such, the significance of cellular speed and spread area is coupled with cellular proliferation and open space availability. In addition, the availability of open space creates a potential for cellular spreading and migration (60). Thus, the potential for spreading and migration was highest following ExoY^+^ infection as gap formation created open space. Although the ExoY^+^-infected cells spread more than the control cells, observing the lowest migration speed in these cells has a greater physical significance and draws attention to ExoY-induced cytoskeletal modifications that limit actin remodeling and cellular migration (50). The next highest potential for spreading and migration was observed following ExoY^K81M^ -treatment. Indeed, the ExoY^K81M^-treated cells were more spread and moved faster than the control cells. The enhanced migration ability indicates the need to examine whether the ExoY^K81M^-induced loose actin bundling facilitates faster migration in future studies (24, 57, 61).

Cyclic UMP-AM during infection with ExoY^K81M^ was able to increase interendothelial permeability and incite pulmonary edema in the isolated lung but was unable to recapitulate the postinfection effects of ExoY^+^ infection, namely decreased cell motility and proliferation. It is unknown how cUMP-AM promotes interendothelial gap formation during ExoY^K81M^ infection without initiating these chronic effects. This discrepancy may arise from the ExoY-mediated signaling provoked via the accumulation of cyclic nucleotides and potentially their synergistic action. During catalytically active ExoY intoxication, cAMP, cGMP, and cUMP are produced together and at different times. It is unknown whether these cyclic nucleotides are needed to act in unison to produce the full effect of ExoY infection; however, here we have shown that cUMP without the other cyclic nucleotides may be sufficient to cause interendothelial gap formation during infection.

After ExoY^+^ infection, there are persistent alterations in cell motility and proliferation which may be in part explained by sustained changes in the actin and microtubule cytoskeleton of the cell. Cyclic nucleotides produced during ExoY^+^ infection cause the collapse of microtubules through the phosphorylation of microtubule-associated tau protein. Further, ExoY is an actin-bundling protein that promotes the accumulation of actin in the cell by subverting actin branching and depolymerization. This interaction with actin remains intact when ExoY is mutated into catalytically inactive ExoY^K81M^. Therefore, ExoY^K81M^ is able to interact with the actin cytoskeleton even though cyclic nucleotides are not produced. In addition, endothelial cells fail to generate actin-rich lamellipodia following ExoY^+^ infection, yet lamellipodia are preserved following ExoY^K81M^ infection. Microtubules are physically linked to actin at their plus end near the plasma membrane where lamellipodia protrusions form to participate in cell migration (62). Further, the microtubule-associated protein tau may be important for actin-microtubule crosstalk (63–65). ExoY^+^ drives tau phosphorylation, leading to loss of tau binding to microtubules, and ultimately, microtubule reorganization and collapse (29, 42, 66). Microtubule collapse disrupts the mechanical forces generated by cellular membranes that create the protrusive force necessary to propel the cells forward (67). The cyclic nucleotide-mediated breakdown in microtubule architecture may explain why and how cellular speed and proliferation are diminished in ExoY^+^-infected but not ExoY^K81M^ + cUMP-AM infected cells after bacteria are removed. Our findings indicate that cUMP is not likely responsible for the sustained disruption of actin- and microtubule architecture. The interaction of ExoY^K81M^ with actin may be a key factor promoting increased cell motility postinfection, as ExoY^K81M^ infection increases cell motility the same as ExoY^K81M^ + cUMP-AM but above cUMP-AM only treated cells. Consequently, future studies are required to better resolve the chronic adaptations that impair cytoskeletal control of cell motility.

A limitation of our study is that we used a membrane-permeable analog of cUMP that may not recapitulate the spatial compartmentation of cUMP produced during ExoY^+^ infection. Although outside the scope of this paper, in future studies we will clarify the role of cUMP and the uridylate cyclase activity of ExoY in the cytosol of PMVECs. In addition, another caveat is that cUMP-AM addition creates a pulse-like signal, and at present, we do not fully understand how cUMP is degraded or extruded. The intracellular concentration of cUMP in single cells is unknown. In the future, we will determine the physiological shifts of cUMP and the consequent locale of PKA activation in PMVECs to the signal transduction role of cUMP during *P. aeruginosa* infection.

Per our investigations, it is evident that cUMP contributes to the development of pulmonary edema during ExoY^+^ infection. These studies are among the first to dissect the cellular signal transduction role of cUMP. For example, baseline cUMP concentrations are similar to those seen with cGMP (31). Endogenous mechanisms responsible for elevations in cUMP are unknown. Yet here, we demonstrate that cUMP is an important interkingdom signaling molecule, allowing bacteria to manipulate the host environment. In the future, it will be important to assess cUMP-responsive proedemagenic effectors that contribute to vascular dysfunction and poor outcomes in hospital-acquired pneumonia (HAP) survivors after *P. aeruginosa* infection.

## DATA AVAILABILITY

Data will be made available upon reasonable request.

## SUPPLEMENTAL MATERIAL

10.6084/m9.figshare.25751457.v1 Supplemental Fig. S1: https://doi.org/10.6084/m9.figshare.25751457.v1.

10.6084/m9.figshare.22825619.v1 Supplemental Video S1: https://doi.org/10.6084/m9.figshare.22825619.v1.

10.6084/m9.figshare.22825670 Supplemental Video S2: https://doi.org/10.6084/m9.figshare.22825670.

10.6084/m9.figshare.22825724.v1 Supplemental Video S3: https://doi.org/10.6084/m9.figshare.22825724.v1.

10.6084/m9.figshare.22825886.v1 Supplemental Video S4: https://doi.org/10.6084/m9.figshare.22825886.v1.

10.6084/m9.figshare.22825913.v1 Supplemental Video S5: https://doi.org/10.6084/m9.figshare.22825913.v1.

10.6084/m9.figshare.22826006.v1 Supplemental Video S6: https://doi.org/10.6084/m9.figshare.22826006.v1.

## GRANTS

This research was funded in part by HL66299 (to T.S. and R.B.), HL66299 Supplement (to A.D.), HL140182 (to R.B. and T.S.), HL148069 (to T.S. and R.B.), HL167997 (to T.S. and R.B.), HL136689 (to C.M.F.), and AI104922 (to D.W.F.).

## DISCLOSURES

No conflicts of interest, financial or otherwise, are declared by the authors.

## AUTHOR CONTRIBUTIONS

A.D., S.S.P., and T.S. conceived and designed research; A.D., S.S.P., and C.Z. performed experiments; A.D., S.S.P., C.Z., C.M.F., and T.S. analyzed data; A.D., C.Z., D.T.T., and T.S. interpreted results of experiments; A.D., S.S.P., and C.Z. prepared figures; A.D., D.T.T., and T.S. drafted manuscript; A.D., S.S.P., C.Z., D.T.T., D.W.F., R.B., and T.S. edited and revised manuscript; A.D., S.S.P., C.Z., C.M.F., D.T.T., D.W.F., R.B., and T.S. approved final version of manuscript.
